# Crafting a compelling curriculum vitae: Navigating the path to professional success

**DOI:** 10.4102/safp.v66i1.5920

**Published:** 2024-03-25

**Authors:** Chantelle C. van der Bijl, Arun Nair, Klaus B. von Pressentin

**Affiliations:** 1 Department of Family Medicine, Faculty of Health Sciences, University of the Free State, Bloemfontein, South Africa; 2 Department of Family Medicine, Robert Mangaliso Sobukwe Hospital, Kimberley, South Africa; 3 Department of Family, Community and Emergency Care, Faculty of Health Sciences, University of Cape Town, Cape Town, South Africa

In today’s competitive job market, your curriculum vitae (CV) is your ticket to securing that crucial job interview.^1^ Crafting a compelling CV is an art that goes beyond listing qualifications; it is about capturing the attention of potential employers. This article delves into the basics of an exceptional CV. Adapt your CV to the specific role you are targeting, highlight your accomplishments effectively and increase your chances of landing the interview that could be the next step in your professional future. You have mastered your fellowship and postgraduate studies. Now, it is time to master constructing a winning CV that unlocks doors to career opportunities.

## Understanding curriculum vitae basics

The CV is a comprehensive representation of your credentials tailor-made for the specific position you are applying for. It can emphasise, accentuate, articulate and persuade the employer that you match the criteria for the position and should give you the interview to open the possibility of getting the job. It is your marketing document and your branding that will help you stand out among the many potential applicants. It should thus be well organised, structured and flow with the advert.

The core sections of the CV are:

1. **Personal information and contact information:** This should include your name (the full name can be seen on the ID enclosed), email address, phone number, mailing address and relevant registration numbers with professional bodies. Other content includes your social media accounts (especially LinkedIn), gender, nationality, race and date of birth.
2. **Education:** This includes all your qualifications, starting from the most recent first, with details of the institution and the date and year the degree was awarded. If applicable, one can include a thesis but be faithful to the information provided and avoid getting caught up with false claims.
3. **Relevant work experience and employment history:** This can be grouped into relevant categories (clinical, research, teaching, administrative, etc.), going from most recent to the past, highlighting essential duties and skills pertinent to the job advert.
4. **Publications:** These can add value to the CV, especially in jobs that are in education and research.
5. **Presentations (oral and posters):** Include professional and conference presentations appropriate to your discipline that can enhance your skill profile.
6. **Honours or awards:** Highlights achievements or skills that show recruiters that others have commended you for your skills and knowledge.
7. **References:** Include these with permission from senior coworkers or supervisors willing to discuss your work-related skills and abilities (both hard and soft skills).

A cover letter can accompany your CV stating why you are interested in the position and supporting how you meet all the criteria to meet the requirements.

## Formatting and style

A practical, aesthetically pleasing CV carefully balances content and style.^2^ The formatting should easily guide the reader through your professional journey, highlighting achievements, qualifications and skills. Try to summarise these as concisely as possible, focussing on relevance and stating them chronologically.

Use clear headings, subheadings and bullet points if the content allows it. Choose a consistent font and size throughout. If your CV is of a clear and professional design, this will enhance essential information and reflect the attention to detail that employers may look for in a candidate.

Consider using an applicant tracking system (ATS)-friendly CV, as increasingly prospective employees use ATS software in online job application screening or selection processes. An ATS scans CVs systematically, identifying specific keywords and qualifications that align with the requirements outlined in the job description.

## Common pitfalls to avoid

Constructing a CV is necessary for anyone’s academic career, yet you are bound to incur some pitfalls in this process. Common errors include:

- Wordiness – overwhelming the reader with unnecessary details may distract them and lead to missing the critical expertise required for the job.
- Sticking to a generic format – candidates may fail to tailor the content to a specific advert or job opportunity.
- Inconsistent formatting and style may distract readers and show inattention to detail.
- An unprofessional personalised email address may deter potential employers.
- Not updating their CV regularly – employers usually want the most current and comprehensive details of your achievements and employment history.
- Not appreciating your growth and success – regularly look at previous versions of your CV. When invited to the interview, highlight this reflection on your professional development.
- Not updating references – Try to have people as references who have recently worked with you and know your most current skills.

A keen eye toward these pitfalls is critical, ensuring your CV is a polished and compelling document that stands out in a competitive job market.^2^ It is helpful to call upon the help of a professional to address the finer points, as this may increase your chances of landing the interview. The South African Academy of Family Physicians’ (SAAFPs) Next5 initiative organised a webinar in 2023 that showcases many of these valuable tips.^3^

In conclusion, a well-written CV is a powerful tool that communicates your qualifications, reflects your personality and underscores your commitment to professionalism. It acts as a dynamic document, showcasing your journey, achievements and unique strengths, making a compelling case for why you are the ideal candidate for a specific role. As the gateway to securing a coveted job interview, mastering the craft of a CV is indispensable in today’s fiercely competitive job market. Vigilance against common pitfalls is crucial for ensuring your CV remains polished and impactful. The CV has the potential to set you apart and significantly elevate your chances of standing out amidst the competition and securing your career opportunities.
